# Acute changes in cardiac structural and tissue characterisation parameters following haemodialysis measured using cardiovascular magnetic resonance

**DOI:** 10.1038/s41598-018-37845-4

**Published:** 2019-02-04

**Authors:** Tushar Kotecha, Ana Martinez-Naharro, Suree Yoowannakul, Tabitha Lambe, Tamer Rezk, Daniel S. Knight, Philip N. Hawkins, James C. Moon, Vivek Muthurangu, Peter Kellman, Roby D. Rakhit, Julian D. Gillmore, Paramjit Jeetley, Andrew Davenport, Marianna Fontana

**Affiliations:** 1 Institute of Cardiovascular Science, University College London, London, UK; 2 Royal Free Hospital, London, UK; 3 Division of Medicine, University College London, London, UK; 4 Barts Heart Centre, London, UK; 5 National Heart, Lung and Blood Institute, National Institute of Health, Bethesda, Maryland USA

## Abstract

In patients with chronic kidney disease (CKD), reverse left ventricular (LV) remodelling, including reduction in LV mass, can be observed following long-term haemodialysis (HD) and has been attributed to regression of LV hypertrophy. However, LV mass can vary in response to changes in myocyte volume, edema, or fibrosis. The aims of this study were to investigate the acute changes in structural (myocardial mass and biventricular volumes) and tissue characterization parameters (native T1 and T2) following HD using cardiovascular magnetic resonance (CMR). Twenty-five stable HD patients underwent non-contrast CMR including volumetric assessment and native T1 and T2 mapping immediately pre- and post-HD. The mean time between the first and second scan was 9.1 ± 1.1 hours and mean time from completion of dialysis to the second scan was 3.5 ± 1.3 hours. Post-HD, there was reduction in LV mass (pre-dialysis 98.9 ± 36.9 g/m^2^ vs post-dialysis 93.3 ± 35.8 g/m^2^, p = 0.003), which correlated with change in body weight (r = 0.717, p < 0.001). Both native T1 and T2 reduced significantly following HD (Native T1: pre-dialysis 1085 ± 43 ms, post-dialysis 1072 ± 43 ms; T2: pre-dialysis 53.3 ± 3.0 ms, post-dialysis 51.8 ± 3.1 ms, both p < 0.05). These changes presumably reflect acute reduction in myocardial water content rather than regression of LV hypertrophy. CMR with multiparametric mapping is a promising tool to assess the cardiac changes associated with HD.

## Introduction

Patients with end-stage renal failure (ESRF) have increased risk of cardiovascular morbidity and mortality, and around 50% of all deaths in patients on haemodialysis (HD) are due to cardiovascular disease^1^. Left ventricular hypertrophy (LVH) and left ventricular cavity dilatation are common findings in patients with ESRF and are associated with increased mortality and cardiac arrhythmias^2–4^. It has been demonstrated that daily HD when compared to traditional thrice weekly dialysis results in significant reduction in LV mass^5,6^. However, interventions to reduce left ventricular (LV) mass do not appear to translate to a reduction in mortality^7^.

It has been suggested that increased afterload and preload in patients with chronic kidney disease (CKD) results in myocardial fibrosis and myocyte cell hypertrophy, and that regression in LVH following chronic HD is due to reduction in both fibrosis and myocyte cell volume^8^. Post-mortem and endomyocardial biopsy studies have both demonstrated the presence of myocardial fibrosis in patients on HD^9,10^. Greater myocardial extracellular water content may also cause increase in LV mass but the contribution and effects of this in patients with CKD have not previously been investigated.

Cardiovascular magnetic resonance (CMR) is now established as the gold standard for quantification of ventricular volumes, mass and function^11^ but it can also provide unique information on tissue composition. CMR with late gadolinium enhancement is uniquely informative for the detection and quantification of myocardial fibrosis in a variety of conditions but the use of gadolinium contrast in patients with ESRF is constrained by concerns about the risk of nephrogenic systemic fibrosis^12^. However, contemporary T1 and T2 mapping methods can surmount some of the shortcomings of non-contrast CMR. Native T1, measured in milliseconds (ms), is increased when the interstitial space is expanded, for example by fibrosis or amyloid deposition, or edema^13–17^. T2 measurements provide an estimate of free tissue water content. For example, T2 is elevated during acute myocarditis and following acute myocardial infarction, and it is proposed that T2 is elevated in the presence of myocardial edema irrespective of myocardial fibrosis^14,17^.

The aims of this study were (1) To assess the changes in myocardial volumes and mass pre- and post-HD and (2) To assess the changes in native T1 and myocardial T2 pre- and post-HD.

## Methods

Twenty-five patients established on thrice weekly HD were recruited at the Royal Free Hospital, London, United Kingdom between October 2017 and June 2018. All participants provided written informed consent. Ethical approval was obtained from the Joint University College London/University College London Hospitals Research Ethics Committee (REC reference: 07/H0715/101). All research-related procedures were performed in accordance with local guidelines and regulations.

### Exclusion criteria

Patients with standard contraindications to non-contrast CMR (e.g. implanted pacemaker, intra-cranial coils, severe claustrophobia, inability to lie flat) were excluded.

### CMR protocol

All CMR scans were performed at 1.5 T (Magnetom Aera, Siemens Healthcare, Erlangen, Germany). Patients underwent non-contrast CMR before and then again immediately after a haemodialysis session. Patients were weighed immediately before each CMR scan. A standard protocol was used that included localizers, cine imaging, native T1 mapping and T2 mapping. Cine imaging (long-axis and short axis stack covering the entire ventricles) was performed using electrocardiographic (ECG)-gated breath-hold steady state free precession (SSFP) or Realtime where the patient was unable to breath-hold or in the presence of arrhythmia. For native T1 and T2 mapping, basal, mid and apical short axis, and a 4-chamber long-axis were acquired after regional shimming. T1 mapping used a Siemens research works-in-progress sequence (WIP1041B) that acquired images using a modified look-locker inversion recovery (MOLLI) protocol using a 5 s (3 s) 3 s sampling scheme: 2 inversions, with images acquired each heartbeat for 5 sec following the 1^st^ inversion, 3 sec recovery, and images acquired for 3 sec following a 2^nd^ inversion^18^. Typical protocol parameters for T1 mapping were: matrix 256 × 144, field-of-view 360 × 270 mm^2^, spatial resolution 1.4 × 1.9 mm^2^, slice thickness 8 mm, flip angle 35 degrees, bandwidth 1085 Hz/pixel, echo spacing 2.7 ms. T2 mapping used the Siemens MyoMap sequence which acquired 3 T2 weighted measurements and performed an exponential fit for each pixel after respiratory motion correction. The imaging used a T2-prepared single shot b-SSFP readout with T2 preparation times (TE) = 0, 25, and 55 ms with a recovery period of 3 heartbeats between measurements. Typical protocol parameters for T2 mapping were: matrix 192 × 108, field-of-view 360 × 270 mm^2^, spatial resolution 1.9 × 2.5 mm^2^, slice thickness 8 mm, flip angle 70 degrees, bandwidth 1184 Hz/pixel, echo spacing 2.6 ms. To assess intra-scan reproducibility, all native T1 and T2 maps were repeated after at least a 5-minute interval in 7 patient scans.

### CMR image analysis

All images were analysed offline using Osirix MD 9.0 (Bernex, Switzerland). The endo- and epicardial borders were manually delineated for each basal-, mid-ventricular and apical short axis map. Obvious image artefacts and coronary arteries were excluded from the regions of interest. Native T1 and T2 were averaged over all three short-axis slices to calculate global averages. Initial analysis of the maps was performed by TK. All mapping images were then independently reanalysed by AMN who was blinded to the initial analysis to assess inter-observer variability. Ventricular cavity volumes and mass were calculated by tracing epicardial and endocardial borders on each end-diastolic short axis cine and endocardial borders on each end-systolic cine. Papillary muscles and trabeculations were included as part of the ventricular mass.

### Statistical analysis

Normally distributed metrics are summarized as mean ± standard deviation and non-normally distributed data are expressed as median (interquartile range, IQR). The paired Student’s T-test was used when comparing parameters between pre- and post-dialysis scans. Correlations between continuous variables were evaluated using the Pearson’s correlation co-efficient. Inter-observer and intra-study reproducibility were assessed using intraclass correlation co-efficient (ICC) with 95% confidence intervals (CI), coefficient of variance (CoV) and Bland-Altman analysis (expressed as bias ± 2 SD for limits of agreement). Reproducibility analysis was performed using MedCalc 13.2.1.0 (Ostend, Belgium). All other statistical analysis was performed using IBM SPSS statistics version 24 (IBM, Somers, New York).

## Results

Twenty-five HD patients (mean age 64 ± 16 years, 17(68%) male) underwent pre- and post-dialysis scans. Baseline characteristics are summarised in Table 1 and full CMR data for each subject is included in the Supplementary File. The mean time between the first and second scan was 9.1 ± 1.1 hours and mean time from completion of dialysis to the second scan was 3.5 ± 1.3 hours. Mean duration of the dialysis session between scans was 3.1 ± 0.8 hours. The median volume of fluid removed in the dialysis session was 2.0 litres (IQR 0.4–2.2 litres), with 4 patients having no net removal of fluid. The mean reduction in weight was 1.5 ± 1.1 kg (Pre-dialysis 76.6 ± 21.8 kg vs Post-dialysis 75.1 ± 21.5 kg, p < 0.001) and mean reduction in body surface area (BSA) 0.02 ± 0.01 m^2^ (Pre-dialysis 1.88 ± 0.28^2^ vs Post-dialysis 1.86 ± 0.28 m^2^, p < 0.001).

**Table 1:** Baseline characteristics.

		Haemodialysis patients (n = 25)
Age (years)		63.9 ± 16.3
Male (%)		17 (68%)
Height (m)		1.68 ± 0.09
Weight (kg)		76.6 ± 21.8
BSA (m^2^)		1.88 ± 0.28
Diabetes		10 (40%)
Hypertension		23 (92%)
Number of antihypertensive agents	0	10 (40%)
	1	11 (44%)
	2	2 (8%)
	3	2 (8%)
Hyperlipidaemia		15 (60%)
Smoking	Current	6 (24%)
	Ex-smoker	7 (28%)
Previous MI		7 (28%)
Previous PCI		5 (20%)
Previous CABG		2 (8%)

BSA, body surface area; CABG, coronary artery bypass surgery; MI, myocardial infarction; PCI, percutaneous coronary intervention.

### Ventricular volumes and mass

**Figure 1:**
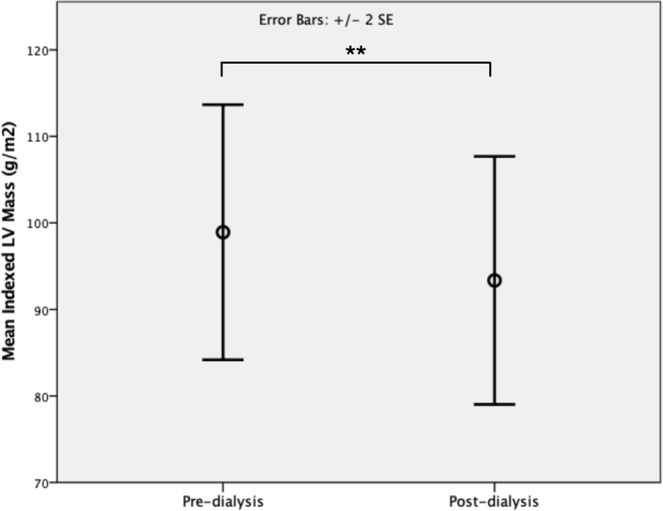
Comparison of pre- and post-dialysis indexed left ventricular (LV) mass. **Denotes p < 0.01.

**Figure 2:**
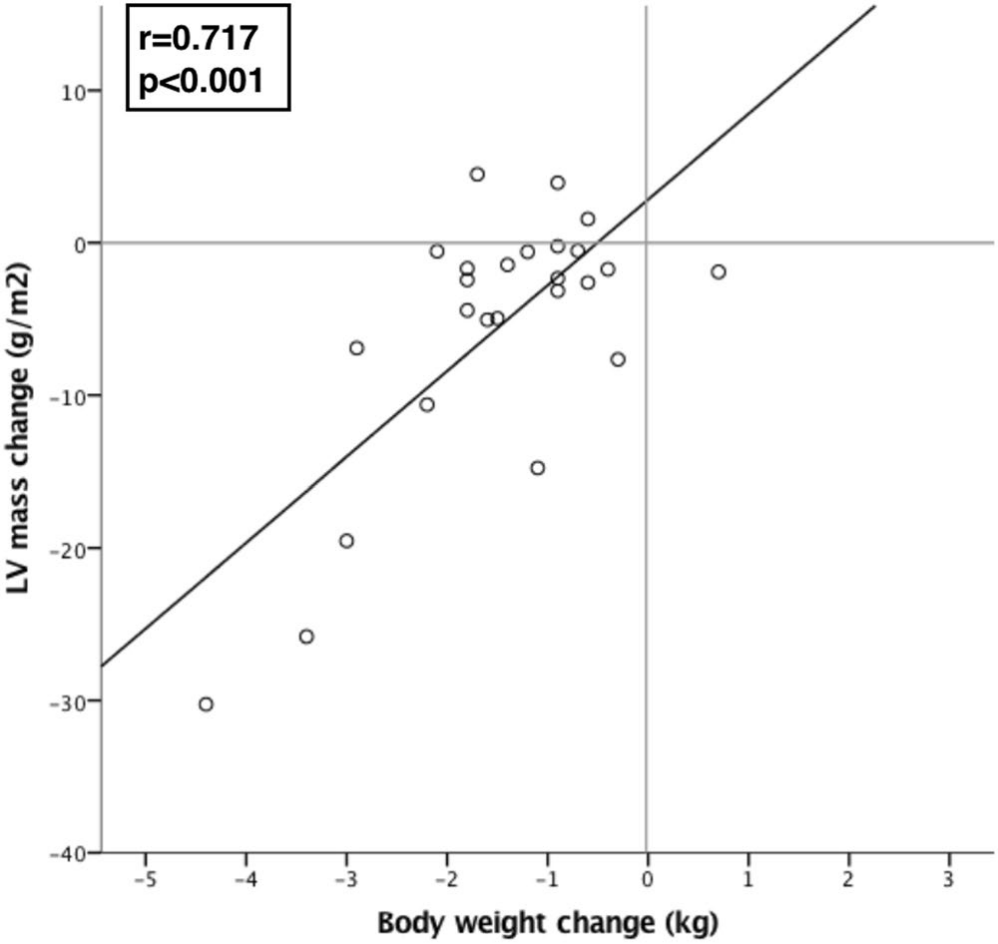
Correlation between change in body weight and change in indexed LV mass following haemodialysis.

Following HD, there was significant reduction in indexed LV mass (pre-dialysis 98.9 ± 36.9 g/m^2^ vs post-dialysis 93.3 ± 35.8 g/m^2^, p = 0.003, Fig. 1) but no significant change in biventricular volumes (Table 2). There was good correlation between change in indexed LV mass and change in body weight (r = 0.717, p < 0.001) (Fig. 2).

**Table 2:** Cardiac structural changes following dialysis.

	Pre-dialysis	Post-dialysis	p-value
LVEDVi (ml/m^2^)	102.8 ± 37.2	100.4 ± 40.1	0.295
LVESVi (ml/m^2^)	50.8 ± 35.3	48.0 ± 33.8	0.092
LVSVi (ml/m^2^)	52.2 ± 15.1	52.4 ± 14.9	0.941
LVmassi (g/m^2^)	98.9 ± 36.9	93.3 ± 35.8	**0.003**
LVEF (%)	54.5 ± 16.5	56.0 ± 14.4	0.149
RVEDVi (ml/m^2^)	91.0 ± 30.8	91.3 ± 32.7	0.895
RVESVi (ml/m^2^)	42.0 ± 25.1	41.1 ± 24.3	0.485
RVSVi (ml/m^2^)	49.2 ± 15.2	50.1 ± 15.0	0.567
RVEF (%)	54.2 ± 17.5	57.0 ± 12.0	0.199

LVEDVi, indexed left ventricular diastolic volume; LVESVi, indexed left ventricular systolic volume; LVSVi; indexed left ventricular stroke volume; LVmassi, indexed left ventricular mass; LVEF, left ventricular ejection fraction; RVEDVi, indexed right ventricular diastolic volume; RVESVi, indexed right ventricular systolic volume; RVSVi; indexed right ventricular stroke volume; RVEF, right ventricular ejection fraction.

**Figure 3:**
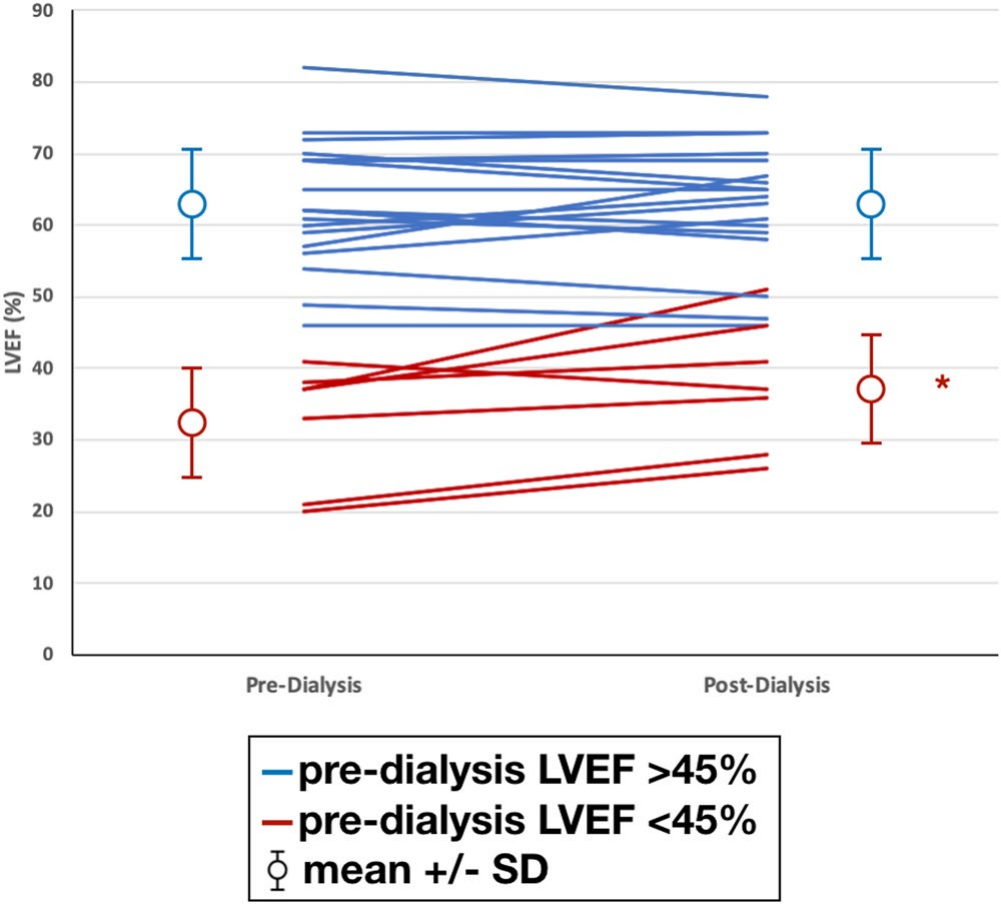
Change in left ventricular ejection fraction (LVEF) following dialysis separated according to baseline LVEF. *Denotes p < 0.05.

In the cohort overall, there was no significant change in LVEF following HD (pre-HD 54.5 ± 16.5% vs post-HD 56.0 ± 14.4%, p = 0.149). However, in patients with impaired pre-HD LV function (LVEF <45%, n = 7) there was significant improvement in systolic function post-HD (change in LVEF: impaired LV function group + 5.4 ± 5.6% vs preserved LV function group −0.1 ± 3.8%, p = 0.01) (Fig. 3). There was no significant change post-HD in RV systolic function or ventricular volumes in patients with impaired LV function compared to those without impaired LV function.

### Native T1 and myocardial T2

**Figure 4:**
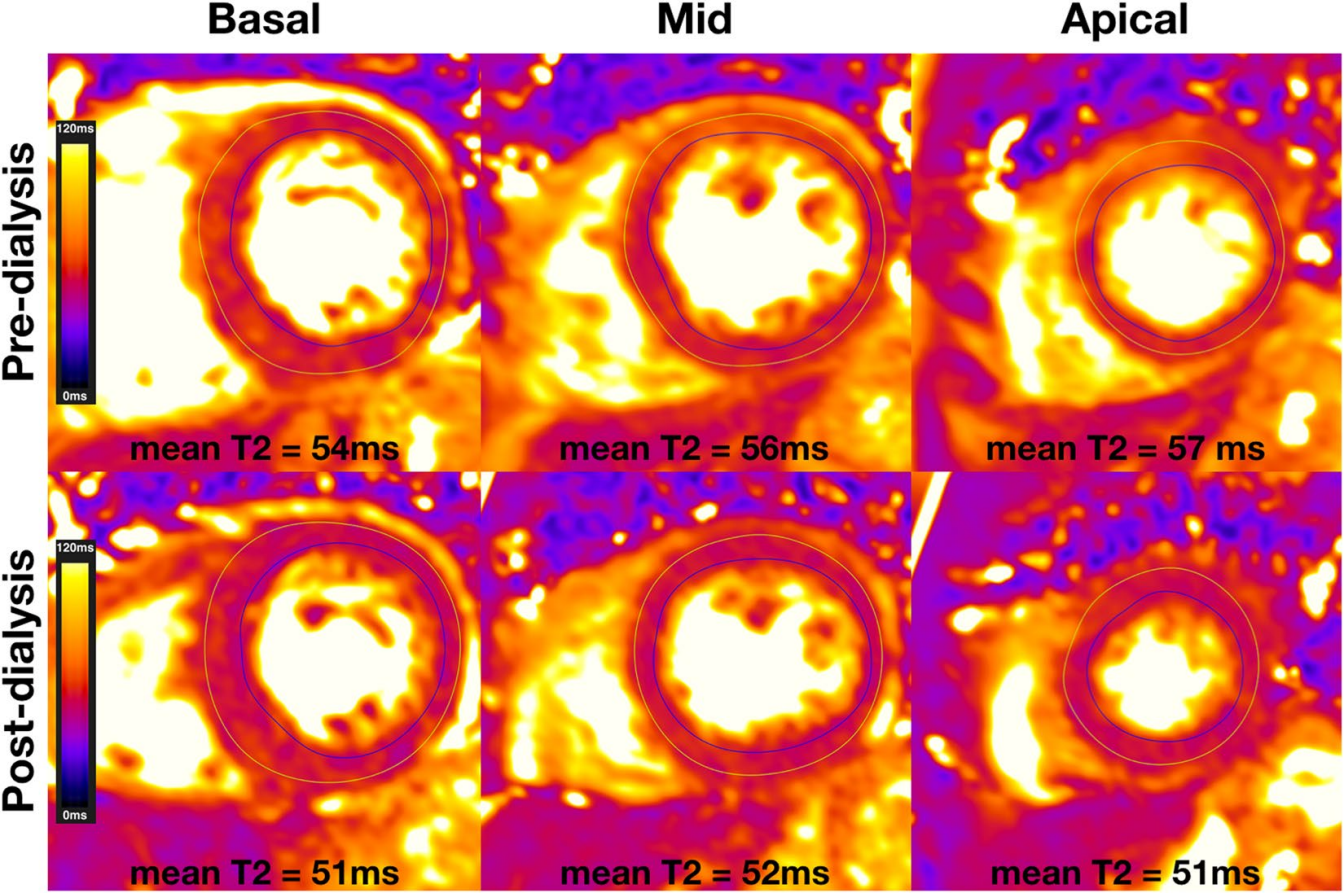
Pre- and post-dialysis quantification of T2. Example of T2 maps of a haemodialysis patient scanned pre-dialysis (top row) and post-dialysis (bottom row). All pre-dialysis images show elevated T2 which is reduced on corresponding post-dialysis images.

**Figure 5:**
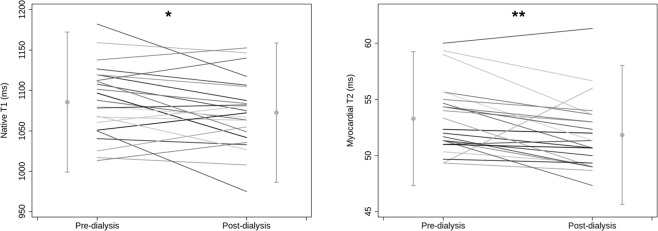
Comparison of pre- and post-dialysis native T1 (left panel) and T2 (right panel). *Denotes p < 0.05 and **denotes p < 0.01.

Both Native T1 and myocardial T2 were significantly lower post-HD compared to pre-HD (Native T1: pre-HD 1085 ± 43 ms, post-HD 1072 ± 43 ms, p = 0.043 and T2: pre-HD 53.3 ± 3.0 ms, post-HD 51.8 ± 3.1 ms, p = 0.006) (Figs 4 and 5).

**Figure 6:**
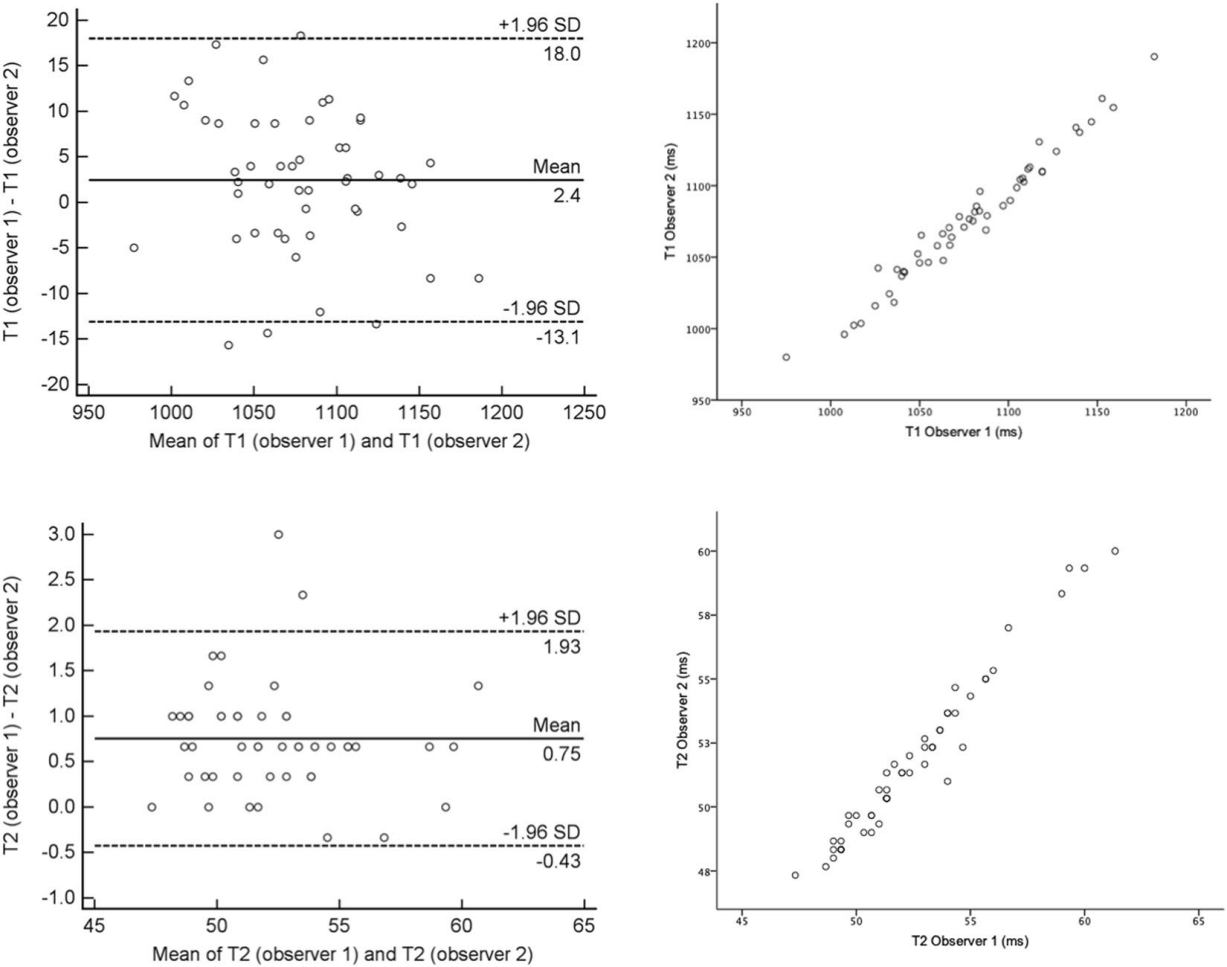
Bland-Altman plots (left) and scatter plots (right) for inter-observer reproducibility of native T1 (top) and T2 (bottom).

The inter-observer reproducibility for both T1 (ICC 0.984 (0.972–0.991), CoV 0.55% (0.44–0.66%), bias 2.4 ms) and T2 mapping (ICC 0.982 (0.968–0.990), CoV 1.32% (1.05–1.59%), bias 0.75 ms) was excellent (Fig. 6). Intra-study reproducibility was also excellent for both T1 (ICC 0.987 (0.927–0.998), CoV 0.36% (0.13–0.60), bias 1.9 ms) and T2 (ICC 0.980 (0.888–0.997), CoV 1.02% (0.35–1.69), bias −0.12 ms).

## Discussion

We demonstrate using contemporary non-contrast CMR techniques that a typical, approximately 3-hour session of HD is associated with significant acute changes in cardiac structure and tissue characterisation parameters. These include reduction in LV mass, T1 and T2, with change in LV mass showing a strong correlation with change in body weight. The changes evident on CMR are likely to reflect acute changes in the patients’ overall fluid status, resulting in reduction of pre-load and myocardial edema.

With the ongoing development of non-contrast CMR mapping, some of the limitations of fibrosis imaging in patients on HD seem to have been overcome. Native T1 has emerged as a potentially useful tool to assess myocardial fibrosis and has been shown to have good correlation with fibrosis as assessed on histology^19,20^. Native T1 is elevated in HD patients compared to controls and the elevation has been attributed to myocardial fibrosis^21^. However other mechanisms can contribute to the increase in native T1, such as the presence of myocardial edema. Patients on HD are exposed to frequent and rapid alterations in fluid status which may be associated with some variable degree of myocardial edema and therefore affect the native T1 signal. T1 elevation in these patients is likely to be due to fibrosis, myocardial edema or a combination of both. However, the acute reduction in native T1 and T2 following HD is in keeping with the hypothesis that some of the elevation in T1 is related to myocardial edema and that HD results in some reduction of the tissue free water content.

In terms of structural changes following HD, there was a significant reduction in LV mass. This reduction correlated well with reduction in body weight following the dialysis session, a surrogate marker for the amount of fluid removed. This observation reinforces the hypothesis that as fluid accumulates between HD sessions, this results in myocardial edema, and that the removal of intravascular fluid during dialysis results in redistribution of this fluid from the myocardium back onto the intravascular compartment.

Several studies have reported reduction in LV mass several months after commencing HD and this has been attributed to regression of myocyte volume or fibrosis^4–6^. The elevation and subsequent reduction of LV mass coupled with the reduction in native T1 and T2 acutely following HD suggests that myocardial edema is the main cause for this reduction and that the elevation in native T1 is a composite of myocardial fibrosis, a well-recognised phenomenon in chronic renal failure^9^ and myocardial edema.

Persistent myocardial edema has a detrimental effect on the myocardium eventually resulting in interstitial fibrosis^22^. It has been demonstrated that over two-thirds of HD patients with LVEF <40% have improvement in LV function to >50% following renal transplant^23^. We propose that the fluid shifts associated with HD provide a recurrent insult to the myocardium resulting in impairment in function. These fluids shifts are removed following renal transplantation. Future CMR mapping studies pre- and post-transplant could provide further insight regarding the mechanisms of this insult. Furthermore, myocardial edema could be one of the mechanisms contributing to the poor prognosis in HD patients, that is not currently explained by the traditional risk factors for cardiovascular disease.

The T2 mapping sequence used in this study was the single shot based SSFP method which is part of the SIEMENS MyoMap product, and the T1 mapping sequence was a works-in-progress MOLLI sequence (WIP 1041B) that allowed for a more flexible sampling strategy with acquisition and recovery periods defined in seconds to further reduce heart rate variability. The technical characteristics of these sequences and comparisons to other methods and protocols have been described previously^24,25^. The T1 mapping approach used in the WIP is based on the original published MOLLI design^18^ with modification of the sampling strategy for reduced heart rate dependence and inversion pulse design for improved inversion efficiency. The inversion recovery based MOLLI approach was chosen over methods employing saturation recovery since it is the most widely available sequence and has improved image quality and precision^26^. The T1 mapping WIP output included both T1 parametric maps as well as estimate standard deviation (SD) maps of precision^27^. The mean SD for the native T1 protocol is approximately 30 ms (per pixel). The product T2 mapping sequence did not provide a SD estimate or goodness of fit, however estimates of the SD for the T2 mapping protocols used in this study are 2–3 ms (per pixel). The precision of the region of interest measurements is improved several-fold over the pixel wise SD depending on the number of pixels averaged. The inter-observer and intra-study repeatability of both T1 and T2 were excellent and comparable to previously published data^28–30^.

### Limitations

This was a small single centre study but is the first to use CMR to assess cardiac changes acutely following dialysis. Dialysis patients included had different aetiologies for their renal failure. Due to the sample size, the effects of this and other potential confounders such as age, gender, hypertension and diabetes were not investigated.

In summary, our findings provide useful information about the acute effects of HD on cardiac function and tissue changes. It is the first to study patients using CMR pre- and post-dialysis sessions demonstrating reduction in LV mass following HD which we attribute to reduction in myocardial edema. Our data suggest that T1 and T2 both fall acutely following a dialysis session and this has implications when dialysis patients undergo repeated follow up scans. When tracking longer term changes (for example over months or years) we would suggest that scans be performed at the same timepoint in their weekly dialysis schedule (for example on the non-dialysis day following the mid-week dialysis session if serial non-contrast studies are performed) and both T1 and T2 should be measured, together with standard structural parameters, to better assess changes in myocardial tissue composition. Furthermore, the acute changes in T1, T2 and LV mass could potentially be used to tailor the HD session to the individual patient, but this would require further large-scale studies.

## Supplementary information

Dataset

## Data Availability

The CMR dataset generated and analysed during the current study is provided in the Supplementary material. Other study-related data is available from the corresponding authors upon reasonable request.
