# Supplementary material for: Acute changes in cardiac structural and tissue characterisation parameters following haemodialysis measured using cardiovascular magnetic resonance
Source: Sci Rep. 2019 Feb 4;9:1388. doi: 10.1038/s41598-018-37845-4 (PMC6362126; doi:10.1038/s41598-018-37845-4)
Supplement: Supplementary file 1 — Dataset [file 41598_2018_37845_MOESM1_ESM.docx]

**Supplementary File**

**Acute changes in cardiac structural and tissue characterisation parameters following haemodialysis measured using cardiovascular magnetic resonance**

**Tushar Kotecha MBChB^a,b^*, Ana Martinez-Naharro MD^b,c^*, Suree Yoowannakul MD^b^, Tabitha Lambe^b^, Tamer Rezk MBBS^b,c^, Daniel Knight MD^a,b^, Philip N Hawkins FMedSci^b,c^, James C Moon MD^d^, Vivek Muthurangu PhD^a^, Peter Kellman PhD^e^, Roby D Rakhit MD^a,b^, Julian Gillmore PhD^b,c^, Paramjit Jeetley MD^b^, Andrew Davenport PhD^b^, Marianna Fontana PhD^b,c^**

*Tushar Kotecha and Ana Martinez-Naharro contributed equally to this work

^a^Institute of Cardiovascular Science, University College London, UK

^b^Royal Free Hospital, London, UK

^c^Division of Medicine, University College London, UK

^d^Barts Heart Centre, London, UK

^e^National Heart, Lung and Blood Institute, National Institute of Health, Bethesda, Maryland, USA

CMR data for each subject
